# Epidemiological and Surveillance Response to Ebola Virus Disease Outbreak in Lofa County, Liberia (March-September, 2014); Lessons Learned

**DOI:** 10.1371/currents.outbreaks.9681514e450dc8d19d47e1724d2553a5

**Published:** 2015-05-06

**Authors:** Koffi Isidore Kouadio, Peter Clement, Josephus Bolongei, Alpha Tamba, Alex Ntale Gasasira, Abdihamid Warsame, Joseph Chukwudi Okeibunor, Martin Okechukwu Ota, Boima Tamba, Nicksy Gumede, Keith Shaba, Alain Poy, Mbaye Salla, Richard Mihigo, Deo Nshimirimana

**Affiliations:** Immunization Vaccine and Emergencies Program, Regional Office for Africa, World Health Organization, Brazzaville, Congo; World Health Organization, Monrovia Office, Liberia; Lofa County Health Office, Ministry of Health and Social Welfare, Voinjama, Liberia; Lofa County Health Office, Ministry of Health and Social Welfare, Voinjama, Liberia; World Health Organization, Monrovia Office, Liberia; Immunization Vaccine and Emergencies Program, Regional Office for Africa, World Health Organization, Brazzaville, Congo; Immunization Vaccine and Emergencies Program, Regional Office for Africa, World Health Organization, Brazzaville, Congo; Immunization Vaccine and Emergencies Program, Regional Office for Africa, World Health Organization, Brazzaville, Congo; Community Health Services Division, Ministry of Health and Social Welfare, Monrovia, Liberia; Immunization Vaccine and Emergencies Program, World Health Organization Regional Office for Africa, Brazzaville, Congo; Immunization Vaccine and Emergencies Program, Regional Office for Africa, World Health Organization, Brazzaville, Congo; Immunization Vaccine and Emergencies Program, Regional Office for Africa, World Health Organization, Brazzaville, Congo; Immunization Vaccine and Emergencies Program, Regional Office for Africa, World Health Organization, Brazzaville, Congo; Immunization Vaccine and Emergencies Program, Regional Office for Africa, World Health Organization, Brazzaville, Congo; Immunization Vaccine and Emergencies Program, Regional Office for Africa, World Health Organization, Brazzaville, Congo

## Abstract

Ebola Virus Disease (EVD) outbreak was confirmed in Liberia on March 31st 2014. A response comprising of diverse expertise was mobilized and deployed to the country to contain transmission of Ebola and give relief to a people already impoverished from protracted civil war. This paper describes the epidemiological and surveillance response to the EVD outbreak in Lofa County in Liberia from March to September 2014. Five of the 6 districts of Lofa were affected. The most affected districts were Voinjama/Guardu Gbondi and Foya. By 26th September, 2014, a total of 619 cases, including 19.4% probable cases, 20.3% suspected cases and 44.2% confirmed cases were recorded by the Ebola Emergency Response Team (EERT) of Lofa County. Adults (20-50 years) were the most affected. Overall fatality rate was 53.3%.  Twenty two (22) cases were reported among the Health Care Workers with a fatality rate of 81.8%. Seventy eight percent (78%) of the contacts successfully completed 21 days follow-up while 134 (6.15%) that developed signs and symptoms of EVD were referred to the ETU in Foya. The contributions of the weak health systems as well as socio-cultural factors in fueling the epidemic are highlighted. Importantly, the lessons learnt including the positive impact of multi-sectorial and multidisciplinary and coordinated response led by the government and community.  Again, given that the spread of infectious disease can be considered a security threat every effort has to put in place to strengthen the health systems in developing countries including the International Health Regulation (IHR)’s core capacities.

**Key words:**  Ebola virus disease, outbreak, epidemiology and surveillance, socio-cultural factors, health system, West Africa.

## INTRODUCTION

Ebola Virus Disease (EVD) or Ebola hemorrhagic fever (Ebola HF) is an acute viral illness, severe and often with case fatality of 90% in humans and nonhuman primates^1^^,^ 2 ^,^ 3 ^,^ 4 ^,^ 5 ^,^ 6 ^,^ 7 ^,^ 8 ^,^ 9 ^,^ 10 ^,^ 11 ^,^ 12 ^,^ 13 ^,^ 14 ^,^ 15 . The first cases in the African Region were reported in 1976 in two simultaneous outbreaks: one in Yambuku community, in Democratic Republic of Congo (DRC), and the other in Nzara community, a remote village of Sudan. Thereafter a total of 24 outbreaks have occurred in the African Region till date, with the last being the most devastating both in terms of its magnitude and impact on the affected countries 7 ^,^ 8 ^,^ 14. The current outbreak has an unprecedented number of over 21,724 cases and 8,626 deaths as of 21st January, 2015.

As part of the response to EVD outbreak in West Africa, several teams including surveillance officers were deployed in the affected countries. A surveillance system is essential to guide the control measures required to reduce morbidity and mortality due to EVD. Surveillance is defined as the ongoing systematic collection, analysis and interpretation of health data essential for planning, implementation and evaluation of health practices. It is closely integrated with the timely dissemination of the data to those who need them^16^. Surveillance can be passive (health facilities based case detection) or active (house-to house case findings). Active surveillance increases the potential to detect cases during an early phase for timely intervention. Both surveillance types can be combined, but this increases the demand for additional human resources and equipment^17^. In summary, surveillance can be defined as "data for action"!

Since the evolution of EVD, in sub-Saharan Africa, few operational studies on this deadly disease have been made available. This paper documents the epidemiology and surveillance outcomes of EVD outbreak in Lofa county of Liberia in 2014. It outlines some lessons learned for use by decision makers, public health officials, the media as well as international and local partners planning and implementing response to future EVD outbreaks.

## MATERIALS AND METHODS

**Background, Location and Population**

The Republic of Liberia consists of 15 administrative counties, with Monrovia as the capital city. There are 88 health districts and 135 administrative districts. See details in map 1. Lofa, the second largest county in Liberia after Nimba is located 241 Km north of Monrovia. The county is composed of 6 districts: Voinjama, Zorzor, Salayea, Foya, Kolahun and Vahun. The population of Liberia is estimated at 4,092,310 inhabitants living on a land area of 111,369 square kilometres^18^ . Liberia is a low income country that encountered several civil wars^19^^,^ 20 ^,^ 21 ^,^ 22 with significant impact on various sectors including: the collapse of the health care system, specifically the diseases surveillance system and the health work force, which contributed to slowing down the achievement of the targets of the Millennium Development Goals (MDG's).

**Figure:**
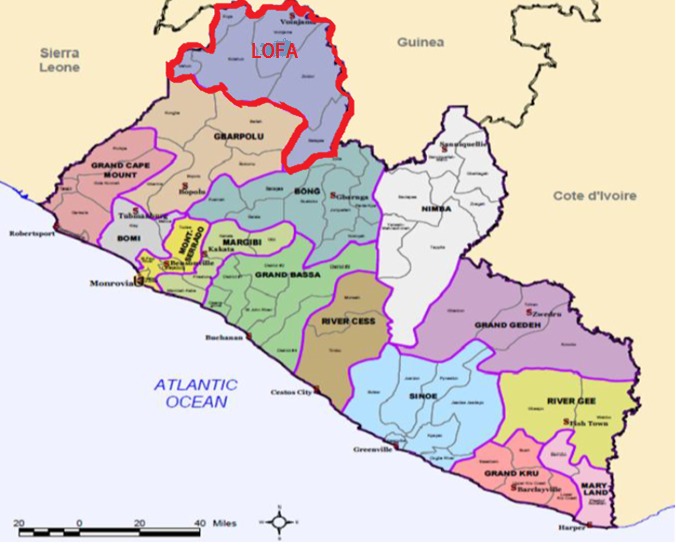
**Map 1: Administrative and Political Map of Liberia**

Suspected cases of EVD were reported in Liberia from March 20th 2014 and the outbreak was confirmed on March 31st 2014. The index case was a Liberian national who was infected in Gueckedou, Guinea and returned for medical care in Borma Foya hospital, Foya district. She died within three days of hospitalization. Cumulatively, six cases were confirmed positive of the virus and all died within the same period. The first wave of the outbreak lasted about forty days.

The index case of the second outbreak was a Liberian woman, married to a Sierra Leonean. On returning from Gbiyedu, Sierra Leone on 23rd May 2014 she was admitted in Borma Foya hospital. After two days of hospitalization, the patient requested to be discharged against medical advice and finally died on 26th May 2014. The corpse was prepared traditionally and taken to Sierra Leone by family members for a funeral. Thereafter, sporadic cases continued to be reported in the district and other parts of the country, including Monrovia.

**Study and Surveillance Period**

The President of the Republic of Liberia declared the EVD epidemic a national health emergency on 26th July 2014^23^ A National Response Plan was launched and a National Task force, which included an Incident Management System (IMS), was established to manage all aspects of the response and to coordinate all technical sub-committees as well as County task force. An Ebola Response Team mechanism was adapted from the National Response Team structure, and established gradually in each District of Lofa County. The components included: (1) Case Management teams; (2) Infection Control and Health Promotion teams; (3) Epidemiology/Surveillance teams; (4) Case Investigation teams (5) Contact Tracing teams; (6) Social Mobilization teams; (7) Burial teams; (8) Media and Communication teams; (9) Logistics teams and (9) Security teams.

The aim of the LOFA Ebola Surveillance Team was to perform both active and passive surveillance in all the districts of the county in order to detect cases at early stage and to take timely and appropriate action. Results of Ebola Emergency Response Team (EERT) in Lofa County, from 20th March to 20 September 2014 (6 months), were documented. The surveillance activities were performed on a daily basis. Cases, deaths and their contacts were reported from the communities and from the Ebola Treatment Unit (ETU) based in Foya, which was one of the few established ETUs in the whole country at that time. In the community, general community health workers (gCHW) were encouraged to perform door-to door case finding and to report to their supervisors, who then report to the District Health Officer and to the County Health Officer for the Response Team. The alert was also provided by individual/household by calling an established phone hotline of the Lofa Ebola ERT based in Voinjama or of the ETU based in Foya. Most of the health facilities were shut down due to an increased contamination and death of health care workers and the associated stigma from the community. On the receipt of an alert by the Ebola ERT, an investigation team is deployed. Suspected or probable Ebola cases were carried to the ETU in Foya for further management (isolation, Lab test and treatment). Swabs were collected from the deceased person for laboratory test. Contacts were followed for 21 days. Different standardized forms were used for case investigation and contacts tracing. All resulting data were kept and maintained in a comprehensive database. Ebola response coordination meetings, held on Tuesdays and Fridays, were chaired by the county superintendent with the active participation of various partners including WHO, UNICEF, MSF, as well as other international and local NGOs**.** Technical meetings were conducted on alternate days. Partners’ meetings were initiated every Saturday, in order to harmonize interventions and avoid duplication of activities as well as to bridge existing gaps.

**Laboratory confirmation and case Managements**

Laboratory tests were initially performed in Gueckedu in Guinea by the European Mobile Laboratory (EMLab) consortium and later in the dedicated laboratory facility established in September by “Medecin Sans Frontiere (MSF)” in Foya. Blood specimen collected from suspected cases and oral swabs specimens from the deceased patients with Ebola-like symptoms were tested using Reverse Transcription-Polymerase Chain reaction (RT-PCR) assay^24^^,^ 25. All confirmed cases were monitored and the patients were treated in the Ebola ETU in Foya. Essential intensive supportive care was provided to patients, including maintaining fluid and electrolyte balance. Ebola prevention and control education were also provided to families whenever a case was detected. Communities and families were encouraged to adopt IPC principles to limit the spread of the disease. These included regular hand washing with plain soap or chlorinated water, assigning only one family member to take care of any person that fell sick, taking due precaution while awaiting the arrival of an investigation team; or immediately bringing the sick relative to the nearest health facility or calling the established hotline.

**Data analysis**

Demographical and epidemiological data as well as Ebola laboratory results were recorded using Microsoft Excel software for analysis.

## RESULTS

As of 26 September 2014, the outbreak affected 5 of the 6 existing health districts in Lofa. The most affected were Voinjama and Foya with more cases (suspected, probable and confirmed), among the female gender. Figure 1 shows that the most affected communities Countywere Barkedu, Jamulor, Womanor, and Samedu located in Voinjama District; Zango town, New Voinjama, Sheriff Quarter and Krakun located in Voinjama district; Kolahun town and Nyonkoitahun located in Kolahun district; and Langbamba, Sorlumba and Kornosue located in Foya district. No case was reported from Salayea district.

**Distribution of EVD cases by districts in LOFA March-September 2014:**
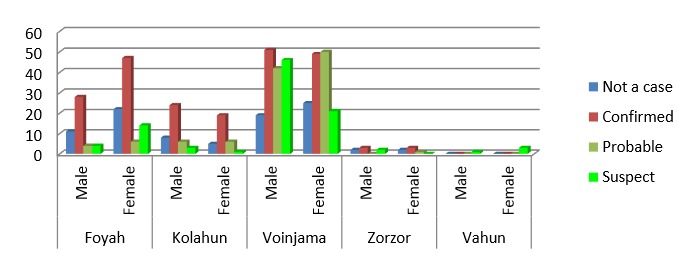

A total of 619 (84%) persons met the cases definitions: with 19.4% (N=143) probable cases, 20.3% (N=150) suspected cases and 44.2% (N=326) confirmed Ebola cases as shown in Table 1. Among the 619 cases, there were more female cases (326; 52.7%) relative to males (293; 47.3%). More deaths occurred among men (51.5%; N=170) than women (48. 5%; N=160). The overall case fatality rate due to EVD was 53.3% (330/ 619). The CFR among health care workers (HCWs) was 81. 8%. The age range of the cases was 1 month to 102 years old and the mean was 31 years old (Figure 2). The group of 20-50 years old was the most affected with the 30-40 years old strata being especially affected. The children, adolescents and young adults (0-20 years old) were more affected than the age group of 60 years and above. Among the 2179 contacts, 1715 completed the 21 days follow up while, 13 dropped out, and 134 developed signs and symptoms and were referred to the ETU in Foya.

**Table 1: Distribution of Ebola Virus Disease cases and deaths by gender March-September 2014:** 

Status	Cases			Deaths		
	Male	Female	Total	Male	Female	Total
Confirmed	136	190	326	88	106	194
Probable	76	67	143	28	19	47
Suspected	81	69	150	54	35	89
Total	293 (47,3%)	326 (52,7%	619 (100%)	170 (51,5%)	160 (48,5%)	330 (100%)

**Distribution of EVD cases by age group in LOFA March-September 2014:**
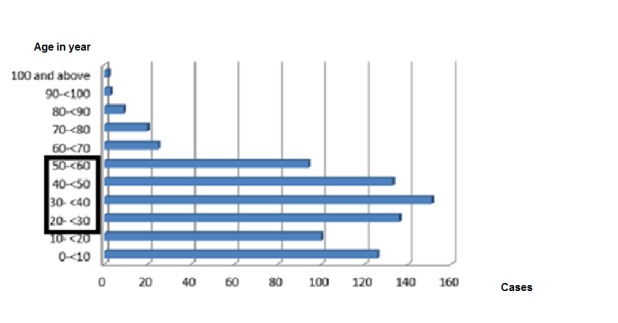

Figure 3 reveals that, the first wave of the epidemic started from 20th March 2014, and the last case reported on 9th April 2014. There were no reported cases until end of May 2014. Cases in the second wave appeared in June increasing rapidly to reach the peak of the outbreak in the second week of August. During this period, the disease spread exponentially reaching other districts (Kolahun, Vahun and Zorzor districts) and was characterized by daily increases in cases and deaths in Lofa County. There was a gradual decline in the disease incidence from end of August until end of September. Then the situation stabilized with some days of no reported cases or deaths.

**Evolution/Epidemic curve of Ebola cases in LOFA country March-September 2014.**
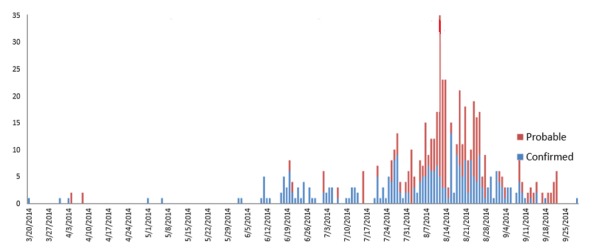

Figure 4 reveals that after the first week of July, the number of cases admitted to the ETU increased daily and peaked in the second week of August 2014. The situation became chaotic from July 27th to August 17th with a steady increase in referrals made to the Ebola ETU in Foya, daily. With the continued effort of the local government health offices, non-governmental organizations (NGOs), the involvement and collaboration of the communities and the strong support of partners (international technical agencies), the daily admission decreased progressively from the third week of August 2014 and reached a very low level at the end of September 2014. By 26th of September 2014, the total number of discharged patients (survivors) from the Ebola ETU in Foya was estimated to 276.

**Trend of daily admissions of patients in Ebola Treatment Unit (ETU) in FOYA, July to 27 September 2014:**
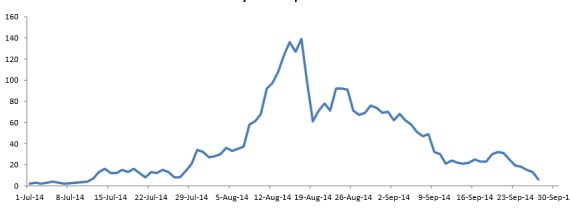

## DISCUSSION

Ebola Virus Diseases (EVD) has been found in nonhuman primates in Asia and United States but human cases are only reported in sub-Saharan Africa since 1976^26^. This may be related to cultural attitude of unsafe preparation of the deceased and/or unsafe burial practices, as well as unsafe handling and consumption of ‘infected’ bush animals.

The evolving outbreak in West Africa is described as the largest EVD outbreak ever recorded for the following 5 majors reasons; (1) the Zaire type of the EVD isolated in West Africa is the most virulent among the 5 types and always associated with high Fatality rate^27^^,^ 28; (2) Before the current outbreak, EVD in human was previously unknown in the West Africa countries. Therefore appropriate preventive and control measures for EVD were unknown to the communities; (3) the EVD outbreak is evolving in low income countries with weak health systems resulting from several years of armed conflicts; (4) population movement within countries in West Africa facilitated the rapid spread of the disease reaching large cities (capital cities) simultaneously; (5) the delay of governments in confirming initial cases and responding to EVD outbreaks.

The WHO declared this outbreak a public health emergency of international concern on 8th August 2014. However, compliance, implementation and monitoring of core capacities of the revised 2005 International Health Regulation (IHR) continues to present a challenge for a majority of partners since the coming into effect of IHR in June 2007^29^. Compliance with the IHR requires that an alert be sent within the required period of 24-48 hours of detection of symptoms. The inability to implement or meet the IHR core surveillance and response requirements, including at designated airports, ports and certain ground crossings in West Africa, specifically in Liberia resulted in the challenge faced in effectively preparing, preventing and controlling, and responding timely to this outbreak. The delay in response to the EVD outbreak was not an issue for the governments only but also for international organizations, which were due to late notification from the affected countries (Guinea, Liberia and Sierra Leone). Furthermore, the affected West African countries had no tradition for coordinated crisis management of the magnitude required during the EVD outbreak. Lofa County was the epicenter, followed by Montserado County where Monrovia (the capital of Liberia) is located. Several factors contributed to the rapid spread of the diseases especially in the initial phase. These included, initial slow response to the epidemic, increased community and household infection, denial, fear and, catering for Ebola related cases in prayer houses, among others.

***Weakness of the surveillance system: ***There were no established surveillance and early warning systems to report such unusual event as early as possible in Liberia. The different operational definitions of Ebola (suspected, probable, confirmed case) and contact cases were not well understood initially.

***The slow response to the Epidemic: ***Misdiagnosis of cases also contributed to the slow response. Health workers often misdiagnosed EVD as malaria, typhoid fever or other diseases especially at the initial stage of the outbreak; and this also contributed to the infection of health care workers who did not implement adequate infection prevention and control measures^31^ . Furthermore, the absence of appropriate/standardized laboratory initially in Lofa County delayed the confirmation of EVD, as well as the appropriate response and early notification to the WHO as recommended^30^. Due to stigmatization and hostility towards workers, most of the health facilities were closed to avoid the potential backlash of the community members who initially viewed them as responsible and the source of propagation of the disease in their community. This closure of health facilities led to the rise and development of other public health problems such as a drop in the treatment of malaria, maternal and child health services.

***Increased community and household infection: ***Many people were infected in the community, especially women and children due to the usual close contact between mother and children. Cross border population movement was an important catalyst of the outbreak. These affected districts in Lofa County sharing border with some affected villages in Guinea. Members of these neighboring communities in Liberia are used to farm, visit relatives and conduct trans-border trade. The most affected age group in our data supports the fact that population movement within and across borders is a major risk factor. Children were exposed through their parents who either succumbed to or survived the disease.

The delay in responding to initial cases allowed the increase for chains of person-to-person transmission. Information flow from peripheral level (the communities) to county level was not timely. The potential of mobile technology in providing data or information^32^ from the community to the central level was limited. When notifications were received, there were delays in reaching far-away communities for investigation and removal of cases and/or deaths from the community. In addition, at the peak of the outbreak in August, there were limited bed spaces in the Ebola Treatment Unit in Foya, and people were being infected while caring for their relatives at home.

***Denial, fear and panic:*** There was denial, misinformation, myths and beliefs about Ebola. It was difficult to reach out and mobilize/ sensitize the affected communities due to over-reaction from the youth and community leaders.

***Hosting Ebola related cases in prayer centers:*** Some religious leaders did not believe Ebola to be a public health problem but rather a spiritual one. Therefore, attempts were made to treat patients through prayers, laying of hands and hugging each other. In this process the pastor, and other members of the congregation became infected with Ebola Disease. This scenario played out in Kornosue, Foya district, where cases and deaths were reported.

***Hostility and safe burials in the community:*** Some of the communities were secretly burying their dead relatives, and involved the ritual washing of Ebola victims that played a significant role in the spread of the infection^33^^,^ 34. However, the exponential increase of cases and deaths in Lofa County caused the community to rethink, leading to a positive change in local practices of caring for the ill and burying the dead. They became more collaborative and more engaged in the fight against EBV outbreak in their community.

***Inadequate community engagement and sensitization:*** It was difficult to reach out and mobilize/ sensitize the affected communities due to over-reaction from the youth groups and other community leaders. This was caused by misinformation, rumors and mistaken beliefs about EVD.

***Contacts running away:*** It was initially challenging to efficiently trace, track, locate and follow-up contact persons. In addition to this situation, stigmatization and denial led suspected contacts to escape to other districts, and even to neighboring countries (Guinea or Sierra Leone) thereby helping to spread the infection.

***Infection among health workers:*** The number of HCWs working at the ETU was less compared to those working in common public and private health centers in the communities, supporting that lack of training on protective measures increases the risk of infection. At the community level, some health workers became infected through involvement in unsafe community treatment of their regular patients. These practices increased transmission in the community after the closure of almost all health facilities in Lofa County.

***Patient’s safety issues: ***The issue of the few persons that tested negative and discharged from ETUs but later developed signs and symptoms in the community is not well understood and requires further investigation. However, it is likely to be related to either timing & sensitivity of the tests or nosocomial infection while at the ETU. This highlights the importance of placing discharged Ebola patients on observation or follow up for 21 days. It was also reported in Voinjama, Lofa that a man who recovered from EVD may have infected his partner later after being discharged from the ETU. In the same vein of minimizing transmission, it is essential to further sensitize discharged men on sexual abstinence or use of other protective methods (condoms) for 3 months, since the virus is reported to remain present in their semen for about 7 weeks^6^.

***Inland and Cross border movements: ***As of August, 2014, the ETU in Lofa, was among the very few ETUs existing in Liberia and the only one in Lofa County. Therefore, people were continually moving from other counties as well as from neighboring countries (villages) to Lofa, seeking treatment. Later on, the persistent efforts of the local government health offices, NGOs, and the strong support of partners (International technical agencies) contributed to gradual development of a comprehensive Ebola response strategy in collaboration with the communities. Under the leadership of the Lofa County authorities, the established comprehensive strategy involving the communities enabled the response team to curtail the epidemic. From the third week of August 2014, we could observe a decrease in number of cases in the community as well as in the ETU throughout the month of September 2014. However, partners treated this stabilization with caution given that more people were coming into Lofa County from other parts of the country coupled with increasing cases being reported in Guinea and Sierra Leone^35^^,^ 36 ^,^ 37. The community of Kpazagizia and Zolowo in Zorzor districts were quarantined for 21 days after a rise in cases, deaths and contacts as a result of increasingly uncontrolled trans-border movement of population between Liberia and Guinea. The hallmarks of the response with the established comprehensive strategy were concentrated on the following approaches:

***Community engagement: ***Local leaders, youth and women associations were involved and able to speak to their communities about EVD. They encouraged their communities to avoid denial, fear and panic and report all suspected cases to the community chief and the District Health Officer (DHO). Community radios continued to play an active role in dispelling myths on Ebola. Alert cases were promptly removed from the community. In the village of Barkedu, local leaders were able to mobilize funds to complement efforts of the CHT in accelerating referral of suspected cases from their communities. A community watch-group was established in all parts of the county, initiating restrictive measures to prevent people from moving from one infected community into another. Some communities were cooperating to report all suspicious persons coming from other parts of the country into Lofa. Town Chiefs and DHOs were informed of any person who entered any community enabling the DHOs through community health volunteers to monitor them. Such suspicious persons with Ebola-like symptoms were also followed up for 21 days. Some church denominations voluntarily organized sensitization/awareness sessions with their congregation. This was intended to discourage other denominations against hiding Ebola-related cases in prayer centers.

***Handling and burial of dead bodies in the communities:*** After further sensitization and acceptance, no community was directly involved in handling and burial of dead bodies. All community deaths were reported to the DHOs for laboratory confirmation to rule out EVD, and conduct safe burial by trained volunteers from the district. These volunteers were selected by the local authorities as a means of respecting the socio-cultural structures of the communities.

***Case management:*** ****By the end of September 2014, Lofa County recorded the highest number of survivors compared to any other counties in Liberia. The staff at the ETU in Foya were primarily local staff from the ministry of health who had undergone regular training by "Medecin Sans Frontiere". This approach built local human resource capacity within the County.

***Referral of suspected cases from the communities:***Communities in Lofa were informed of hotlines (ETU in Foya and the DHOs in Voinjama) to enable them call for prompt help. The response from the ETU and DHOs to urgently investigate these cases and refer them to Foya was swift.

***Contact tracing:*** Given regular refresher trainings of the contact tracing teams, very few contacts were lost to follow up (approximately 0.3%). Contacts were confined to their localities and advised to restrict movement within the community.

***Coordination, logistics and finance: ***All districts had their taskforces chaired by district commissioners or district superintendents. Challenges were discussed during coordination meetings and action points were followed up and implemented. Collaboration between Lofa County, district authorities, other line Ministries and partners with the ministry of health and social welfare in all aspects of the Ebola response was encouraging.

***Social Mobilization and communication:*** Advocacy meetings were held with all district authorities, local, religious and traditional leaders. In communities considered to be resistant to implementing the IPC measures, a particular focus was placed on stigma and community protection as well. Community mobilization, sensitization and health promotion activities were focused on the most affected communities based on the Ebola weekly surveillance report provided during the biweekly Ebola response coordination meetings. All communication outlets including local community radios, interpersonal communication, posters, focus group discussions and testimonies from Ebola survivors were used to mobilize the communities.

***Strengthening health system for effective response: ***This outbreak shed light on the weaknesses of the several components of the health system of the affected countries. In prevention of future outbreaks it is important to addresses the weaknesses in human resources for health, service delivery etc, in a concerted and systematic way to rebuild local health systems. A strong health system will not only detect and curtail epidemics on time but protect against other killer diseases and improve the health of the population in general. Furthermore the creation of a larger and more dynamic emergency response fund such as that of the African Public Health Emergency Fund guarantee a ready fund that would facilitate a more robust and prompt response should such epidemics and emergencies occur in the future^38^.

***Limitations of the paper:*** Firstly, EVD was unknown, misdiagnosed and the response was initially inappropriate. Therefore, cases and their contacts at the beginning of the outbreaks may have not been well captured in the database. Secondly, Ebola cases and deaths in the communities may not have been accurately reported and recorded in our database since some communities were hiding cases and making secret burials. Thirdly, our analysis was restricted to Lofa County and the period limited to the first 6 months of the response. With these limitations, the paper may not provide the most complete picture of EVD outbreaks in Lofa County. However, it documents some interesting aspects of EVD epidemiology and prevention and provided insights for future outbreaks response and control.

## CONCLUSION

The experience from Lofa County, Liberia highlights that socio-cultural factors, lack of community participation and weakness of the health systems are risk factors for propagating epidemics. A number of lessons learnt are highlighted that could be helpful in guiding interventions should such or similar epidemics occur. The involvement of local authorities, community leaders and civil societies, and other partners working in synergy made a remarkable and positive impact in the response to the EVD outbreak. This outbreak demonstrates that in this interconnected and globalized world, the spread of infectious disease can be considered a security threat. Therefore WHO, Member States and partners need to continue working collectively to bridge identified gaps in IHR core capacities in the most efficient and effective way, using existing strategic approaches, networks and resources. However, more studies are needed on the impact of EVD on the countries’ economy, agriculture, security, culture, education (school closure), as well mental & psychosocial wellbeing. It may be important to study the global amount of resources that were needed (from all partners involved) to mount the response of EVD outbreak in Lofa county, including the community engagement.

## Competing Interests

The authors have declared no competing interests.

## Corresponding Author

Joseph Chukwudi Okeibunor (okeibunorj@who.int)
